# Bored to Be Wild: How Boredom Is Related to Pre-Service Teachers’ Intention to Persist in Their Studies

**DOI:** 10.3390/ijerph18094452

**Published:** 2021-04-22

**Authors:** Catherine Audrin, Marine Hascoët

**Affiliations:** 1 Media, Digital Use and Informatic Didactics Teaching and Research Unit, Lausanne University of Teacher Education, 1007 Lausanne, Switzerland; 2 Swiss Center for Affective Sciences, 1202 Geneva, Switzerland; 3 Child to Adult Development Teaching and Research Unit, Lausanne University of Teacher Education, 1007 Lausanne, Switzerland; marine.hascoet@hepl.ch

**Keywords:** boredom, control-value theory, academic intention to persist, pre-service teachers

## Abstract

Boredom is an emotion that often arises in an educational context. Past research suggests that boredom depends on specific cognitive appraisals, such as how people can control the task and how much they value it. Research further suggests that boredom is related to negative academic outcomes such as lower grades and a higher risk of dropping out. Here, we tested a mediation model on 324 pre-service teachers during the first lockdown of 2020 in Switzerland to assess (1) how control and value predicted boredom, and (2) how boredom was related to the intention to persist at university. We hypothesized that (1) the more participants felt lacking in control and low in value, the higher their boredom and (2) the more intense their boredom, the lower their intention to persist. We further hypothesized that both control and value would be positively related to the intention to persist, and this link may be mediated by boredom. Our results provide partial support for our mediation model as we found a significant indirect link between control and intention to persist through boredom. More specifically, the more participants lost control over their studies, the more they felt bored, which in turn was negatively related to their intention to persist.

## 1. Introduction

Academic emotions are defined as emotions which are experienced by learners in educational settings, and which are directly related to academic learning, classroom activities and learning achievement [1,2]. Academic emotions notably include anxiety, pride, surprise, and boredom.

In this study, we are interested in academic boredom during the COVID-19 pandemic. During this specific period of time, all courses were taught online, which may increase students’ boredom. More specifically, we want to test how participants’ perception of control and importance (value) of their studies may predict boredom, which in turn may predict their intention to persist. In the following paragraphs we will first define academic boredom and describe one of the prominent theoretical frameworks focusing on academic emotions.

### 1.1. Academic Boredom 

Academic boredom is typically experienced when completing homework or performing learning exercises in class [3]. Although the numbers vary, boredom is thought to be a frequently felt emotion in academic settings, as 40 to 59% of university students report feeling bored [4]. Boredom is defined as an “unpleasant feeling, [with] lack of stimulation and low-arousal” [5], p. 532. This emotion is characterized by multiple components such as affective (negative feelings), cognitive (perception of time passing slowly), motivational (willingness to change the situation), physiological (low arousal), and expressive (both facial and postural expressions) [6].

Literature highlights several important specificities of boredom. First, boredom is not “simply” a lack of interest [3]. Indeed, while a lack of interest is neutral in terms of valence, boredom is related to negative affective states. Second, due to the difference in valence, interest and boredom have differential motivational consequences; while lack of interest does not imply neither an urge to change nor to engage in the situation, boredom is related to an impulse to escape the situation [5]. More specifically, functional theories regarding boredom suggest that this emotion not only urges the individual to disengage from the task they are in, but also to look for alternatives [7,8]. As highlighted by Bench and Lench [8], people will look for novel alternatives which allow a change of one’s affective state. As such, boredom enhances one’s motivation to look for new goals [9]; when experiencing boredom, people feel that a change in behavior is needed, they will look for a situation which is different than the current one. More specifically, experiencing boredom will prompt individuals to look for a change from a situation perceived as uninteresting or non-stimulating to a situation perceived as interesting and in line with their plans [9]. Finally, boredom has a central set of features which are not explainable by any other emotion, such as the feeling of being unchallenged and of perceiving one’s activity as meaningless [10]. 

### 1.2. The Control-Value Theory

Academic boredom has been studied through the appraisal theories framework, which proposes that cognitive appraisals are at the source of emotions, and that appraisals cause and constitute emotional experiences [11,12]. More specifically, the control-value theory (CVT) is a central theoretical framework widely used in the literature to study academic emotions [13]. The CVT posits that there are two individual determinants of academic emotions, and boredom in particular [14]. Academic boredom arises (1) when students perceive the activity either as over-challenging or under-challenging (control appraisal) and (2) when students do not value the task that they are working on, i.e., if they perceive it as meaningless, useless or irrelevant (value appraisal [13,15]). The control appraisal refers to how people perceive that they have causal influence over their actions and outcomes, while the (subjective) value appraisal refers to the valence of these actions and outcomes [5]. Note that boredom has more often been reported to arise when control appraisal was low [5]. 

The CVT provides insights on how and why boredom may have detrimental impacts on students’ academic achievements, but also on cognition, motivation and learning strategies [16]. First, authors suggest that feeling bored focuses learners’ attention on their emotional experience instead of leaving their cognitive resources free for the academic activity. Second, as we saw earlier, boredom is related to an urge to change activity, it is expected to diminish students’ intrinsic motivation and persistence in learning. These two elements may further relate to a tendency to adopt shallow experience strategies, as learners have lower cognitive resources and intrinsic motivation, they will less often use meta-cognitive strategies in learning and adopt more superficial learning strategies [16]. 

Research has highlighted numerous detrimental effects of boredom on students’ academic lives in many different grades such as primary school [17], secondary school and universities [18]. Notably, numerous studies emphasize that boredom is related to higher attentional problems [5], lower lecture attendance [19], lower effort regulation [5], lower intrinsic and extrinsic motivation to learn, which in turn erodes academic achievement [16,20,21]. Moreover, as highlighted by Daschmann et al. [22], boredom is positively correlated with lower grades, enhanced absenteeism, and a higher risk of dropping out. 

### 1.3. Persistence in Higher Education in the Light of Expectancy-Value Theory

Persistence, intention to persist and dropping out in higher education are of great interest. Intention to persist is considered as a proximal measure of actual persistence [23]. In the rest of this paper, we will use both terms interchangeably. By focusing on understanding the processes that lead students to persist, it is possible to better identify at-risk students, support students’ academic trajectory and help university staff to prevent students from dropping out [23]. This topic is particularly important in the field of teacher training in Switzerland; according to forecasts, the numbers of students will increase twice as much as the number of teaching staff, which will lead to a shortage of teachers [24]. 

In the context of university, persistence is a complex process and depends on many factors, such as family background (e.g., SES level), individual attributes (e.g., gender), pre-university achievement, as well as institutional and social experiences [25,26,27]. Persistence has also been related to parental support, level of stress and depression, first year grade point average and performance goals [26,28,29]. 

The expectancy-value theory [30] can enlighten us on the processes that lead students to persist. According to this theory, achievement-related choices and performance in a task are influenced by two variables: the expectation of success (i.e., the more the person thinks he or she can succeed in this task, the better their engagement, persistence or performance) and the subjective task value (i.e., the more the task is important or useful for the person, the better their engagement, persistence or performance). Drawing on this theory, research [31] has found that the expectation for success and subjective task value in math and language courses influence performance, career intention and academic aspiration for primary and secondary school students. In the university context, a study [32] showed that the expectation for success and subjective task value encourage choice, effort, persistence and continuation in engineering for undergraduate engineering students. In the same way, Neuville et al., [21] showed that the intention to persist in the first year of university was influenced by students’ perception of their probability of success and by the value and the interest they had for their studies. Neuville et al., [21] further revealed that students who had a negative emotional adjustment (i.e., a negative emotional state like feelings of distress or a negative physical state like a somatic problem) showed a lower intention to persist. However, to the best of our knowledge, no research has studied the role of specific emotions with the intention to persist in university.

### 1.4. The Specific Context of COVID-19 Lockdown

Although several studies have recently focused on the importance of boredom within the context of COVID-19, most of these focused on the impact of boredom on psychological outcomes such as psychological and emotional distress [33,34], perception of time [35], the adherence of measures such as social distancing [36,37] or containment [38]. Several studies have focused on students’ emotions in the COVID-19 context [35] as well as on the impact of online technologies on student engagement [39], but no study has yet focused on the potential impact of academic boredom on students’ intention to persist in their studies in the COVID-19 context. In this study, we are interested in how Swiss pre-service teachers’ boredom is related to their intention to persist in their studies during the first lockdown of March 2020. During this specific period of time, all courses were taught online, which might increase the risk of dropping out [40,41,42]. It is possible that dropping out is not related to the love of the profession but is partly due to how students feel in class. In this particular context, it is therefore essential to understand the factors that can undermine the persistence of pre-service teachers in their studies.

### 1.5. Hypotheses

Among the numerous factors at stake, we focused on control and value, and how these appraisals were related to boredom and the intention to persist. More specifically, we hypothesized, in line with the CVT, that (1) control and value appraisals would negatively predict boredom and (2) that boredom would, in turn, negatively predict the intention to persist.

Moreover, as highlighted by Pekrun [13], subjective control is referred to as “the perceived causal influence of an agent over action and outcomes” [13], p. 317. Thus, control depends on subjective expectancies and attribution implying appraisals of control, which can be directly related to Eccles’ [30] definition of expectation of success. Regarding the value appraisal, it is defined as “the perceived valences of actions and outcomes” [13], p. 534. As further explained by the CVT, perceived value refers to how important the task and its potential outcome are to the individual. In this sense, the concept is really close to the subjective value defined by the expectancy-value theory. Research based on the expectancy-value theory has shown that the expectation of success and the task value are closely linked to achievement-related choices and persistence [27,28]. Therefore, in line with the expectancy-value theory, we further hypothesized that both control and value would be positively related to the intention to persist, and this might be mediated by boredom.

## 2. Materials and Methods

### 2.1. Participants and Procedure 

Participants were comprised of 324 students from the University of Teacher Education (222 female). Students were recruited to participate in an online survey and the return rate was 40.5%. Of the participants, 153 students (47%) were studying at the bachelor degree level, while the rest of the sample (171 students, 53%) were studying at the master’s degree level. Note that all participants (both bachelor and master’s degree level students) were pre-service teachers and they all had to complete internships during their studies. Before starting the completion of the online questionnaire per se, participants were first asked to give their consent to participate in the study. Participants were then asked to answer self-reported questionnaires designed to measure (1) control appraisal, (2) value appraisal, (3) boredom and (4) an intention to persist. The order of the items was randomized between participants. 

### 2.2. Materials and Methods 

To measure the control appraisal, we used the scale of Lardy et al. [43], which was designed to measure participants’ perceptions of competence. The scale consisted of three items such as: “I am fully capable of completing my semester”, “When I need to learn something new, I’m pretty sure I can do it”, and “I feel I am able to perform well in important subjects” (alpha = 0.81). All items were measured on a scale ranging from 1 (strongly disagree) to 7 (strongly agree). 

To measure the value students gave to their studies, we used the scale of Neuville et al., [23], which was validated in 2004 [44], and is adapted from Eccles’ research [30]. This scale had an acceptable reliability (alpha = 0.69). This scale comprised of five items such as: “The risk of failure scares me”, “It is important for me to prove to myself that I am capable of succeeding in my year”, and “It is important for me to get a good grade on the exams”. All items were measured on a scale ranging from 1 (strongly disagree) to 7 (strongly agree). 

Boredom was measured using the scale of Neuville et al. [23], which was initially designed to measure students’ intrinsic interest toward a task (7 items). As highlighted earlier, boredom is not a “simple” lack of interest. Thus, we kept items that (1) emphasized negative feelings embedded in boredom such as: “The way the teachings are given is boring”, “I don’t really appreciate the lessons of my curriculum”, and (2) highlighted the cognitive aspect of boredom, which was the perception of time passing slowly such as: “I have to force myself to pay attention to the courses/seminars”. The scale presented acceptable reliability (alpha = 0.73). Items were measured on a scale ranging from 1 (strongly disagree) to 7 (strongly agree).

Intention to persist in the university was measured using the scale used by Neuville [23,44]. Intention to persist can be considered as a proximal measure of actual persistence [23]. This scale consists of eight items such as: “Even if I didn’t pass this year, I would start the same studies all over again”, and “With two months of hindsight, I am satisfied with my choice”. Four items were reversed, such as, “If I had known at the beginning of the year what I know now, I would have made another study choice”. The scale presented acceptable reliability (alpha = 0.85). Participants were asked to answer on a scale ranging from 1 (strongly disagree) to 7 (strongly agree).

### 2.3. Data Analyses 

**Figure 1:**
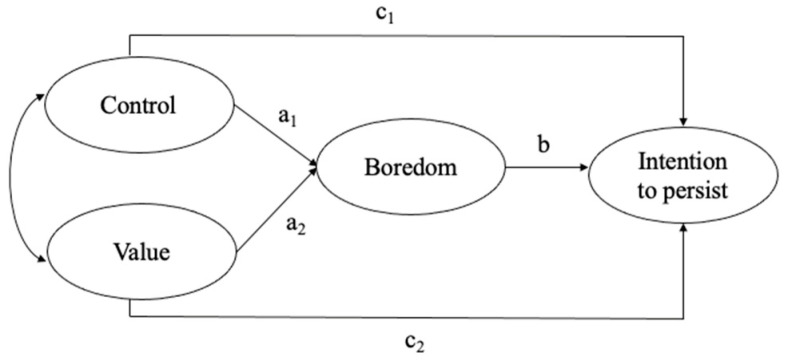
Hypothesized model. a1, link between control and boredom; a2, link between value and boredom; b, link between boredom and intention to persist; c1, link between control and intention to persist; c2, link between value and intention to persist.

All analyses were performed on R (R Foundation for Statistical Computing, Vienna, Austria) using the lavaan package [45]. We performed a mediation analysis with structural equation modeling (SEM) to test (1) how control and value predicted boredom, and (2) how boredom was related to the intention to persist at university. We hypothesized that (1) the more participants felt a lack of control and low in value, the higher their boredom and (2) the more intense their boredom, the lower their intention to persist. We further hypothesized that both control and value would be positively related to the intention to persist (see Figure 1).

For each latent factor, all items were kept as their loadings were equal to, or higher than, 0.40. To assess the model’s goodness-of-fit, we used specific indices which had different measurement properties, as recommended by Hu and Bentler [46]. First, we reported on Chi^2^ and its significance. As it is significant, this suggests that something may (locally) be problematic. Thus, we tested potential local misspecification using the miPowerFit command implemented in the semTools package [47]. None of the parameters were considered as misspecified. We further used the standardized root mean square residual (SRMR), the root mean-square error of approximation (RMSEA), the comparative fit indices (CFI), and the Tucker–Lewis index (TLI). Models with RMSEA with values up to 0.08 reflected reasonable errors of approximation [48]. This value was also considered as a good fit for the SRMR [48]. Regarding the CFI, scholars generally acknowledged that a value greater than 0.90 reflected an acceptable distance to the perfect fit. Finally, the TLI indicated how the model of interest improved the fit in relation to the null model, and a TLI value equal to, or greater than, 0.90 reflected an acceptable distance to the perfect fit. Standard errors were computed using the robust method, and we used a bootstrap based *p*-value. Indirect effects were computed following Hayes [49,50]. More precisely, the direct effect was computed as the direct link between the intention to persist and control (c1) and value (c2). We further defined the link between control (a1), value (a2) and boredom as the mediator effect, and the link between boredom and the intention to persist as (b). The indirect effect through control was thus the product of a1 × b and the indirect effect through value was the product of a2 × b. The total indirect effect was computed as the sum of a1 × b + a2 × b and the total effect was c1 + c2 + a1 × b + a2 × b. 

## 3. Results

Descriptive analyses presented in Table 1 suggest that pre-service teachers have a high intention to persist (M = 5.93), and that they value their studies (M = 5.77). They also have a moderate level of boredom (M = 4.08).

These results further suggest that control is moderately correlated to boredom (*r* = −0.26, *p* < 0.01) and to the intention to persist (*r* = 40, *p* < 0.01), but value is not (respectively, *r* = −0.10, *ns.* and *r* = 0.05, *ns.*). Moreover, boredom is significantly correlated to the intention to persist (*r* = −0.27, *p* < 0.01). 

The model provided good fit (SRMR = 0.06, RMSEA = 0.06, CFI = 0.92, TLI = 0.90, Chi^2^(127) = 288.39, *p* < 0.001, Chi^2^/df = 2.271).

**Figure 2:**
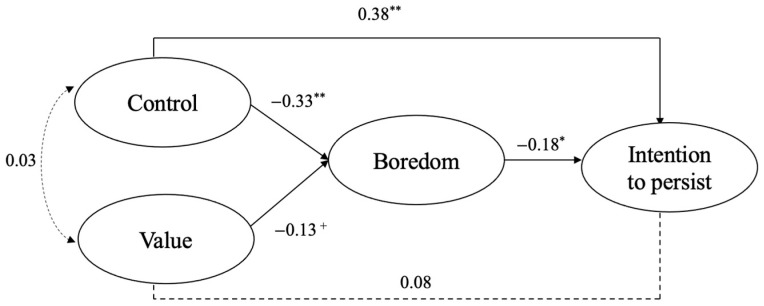
Results of the mediation model: +, *p* < 0.10, *, *p* < 0.05, **, *p* < 0.01.

Graphical depiction is provided in Figure 2. Factor loadings are reported in Table 2, regression coefficients in Table 3 and the direct and indirect effects in Table 4.

As hypothesized, our results reveal that low control negatively predict boredom (*β* = −0.33, *p* < 0.001). This is also the case for value, but marginally (*β* = −0.13, *p* = 0.09). The more participants feel low in control and value, the more they feel bored. Our second hypothesis also found support as our results revealed a negative link between boredom and the intention to persist (*β* = −0.18, *p* < 0.01). However, our last hypothesis was only partially supported. While high control positively predicted an intention to persist (*β* = 0.38, *p* < 0.001), this was not the case for value (*β* = 0.08, *ns*). While our results highlight a significant total indirect link between control, value and persistence, this is mainly driven by the significant link between control and intention to persist through boredom. 

## 4. Discussion

The purpose of this study was to assess how boredom was related to persistence at university. Data were collected on 324 pre-service teachers in Switzerland. Descriptive results first revealed that students’ intention to persist was high, while boredom was moderate. Moreover, our results provide support for the control-value theory as they highlight a significant negative link between control, value and boredom. These results are in line with previous evidence highlighting the conditions in which academic boredom may arise (i.e., when students perceive that they have low control on the task, as well as when they feel that the task is meaningless or useless). 

Moreover, our results provide partial support for the expectancy-value theory. Our results revealed a significant link between control (i.e., expectancy of success) and students’ intention to persist in their studies. This result is in line with Neuville et al.’s results [23]. However, contrary to Neuville and colleagues, we could not find a significant link between subjective value and the intention to persist. The students interviewed were in work–study programs, and it is possible that their intention to persist in their studies has more to do with the value they place on their internship than with the value they place on the academic curriculum. This hypothesis needs to be tested in future studies.

Finally, mediation analysis highlighted an indirect link between control and the intention to persist through boredom; the more students perceive their loss of control, the more they are bored, which in turn diminishes their intention to persist in their studies. Such results thus highlight the importance of emotions felt in an academic context; there is not only a significant direct link between cognitive appraisal and the intention to persist, but appraisals are also directly related to the intensity of academic boredom, which in turn is negatively related to the intention to persist. 

While we focused on Pekrun and colleagues’ [5] control and value theory to predict boredom, other models such as the meaning and attention (MAC) model describes when boredom arises [51]. The MAC model more specifically suggests that boredom occurs (1) when people are not able to engage their attention in a given activity (attention component) and (2) when they perceive this activity as low in meaning (meaning component). Stated differently, boredom occurs when people feel (1) unable and (2) unwilling to engage in the cognitive activity. We believe that the meaning component (the “(un)willingness” to engage) may be closely related to the value appraisal emphasized in the CVT. Indeed, as described earlier, value appraisal refers to how important the task and its potential outcome are to the individual. Regarding the attentional component, to a certain extent it may be related to the control dimension. As defined in the MAC model, the attention component refers to how people feel they can be cognitively engaged in the task. By comparing the perceived resources and demands, the attention component allows a subjective evaluation on how people feel they are able to handle the demands of the task. Control appraisal depends on subjective expectancies and attributions implying appraisals of control, and refers to how people feel they have a causal influence over their own actions and outcomes. Attention and control appraisals may share some common features: both tap into the subjective evaluation on how one is able to handle the situation. However, we believe that the control appraisal focuses more on how people feel that they can have an impact on the situation, while the attention component refers to the perceived balance between one’s resources and the task’s demands. In that sense, attention and control appraisal have different meanings. We believe that future studies may integrate both theoretical frameworks to predict boredom and the intention to persist in academic settings. It might be interesting to assess how control and attention are related with each other and how they predict boredom. 

Past studies show that persistence at university is a complex phenomenon with many determinants [23,24,25]. However, to our knowledge no studies have focused on the role of negative emotions in this process. This study therefore makes a contribution by showing that boredom can influence intention to persist. As our results reveal a significant link between boredom and the intention to persist, a promising line of research is to assess how students may cope with boredom [52,53,54], so that it may prevent the negative consequences of being bored in an educational context. 

Interestingly, several studies have suggested that boredom may have positive impacts [3,55,56,57]. Notably, as boredom may act as a signal for individuals to make changes [7], it may foster creativity [58]. Future studies should dig deeper into the positive impact of boredom on academic outcomes and in order to distinguish which personal or situational characteristics may foster positive (vs. negative) impacts of boredom.

A primary limitation of this study is that we focused exclusively on proximal antecedents of boredom. However, more distal factors (such as learning environment or teacher-perceived emotions) are of importance too [21,59]. A second limitation refers to the measures we used. The measure of boredom specifically focused on study-related boredom, where boredom may be related to course, class, task or test contexts [53]. The measure of control mostly focused on how participants felt they were confident with their potential success in their studies. While these items captured the “control” dimension defined in the expectancy-value theory, they showed two limitations: first, they did not capture the whole concept of “control”. As defined by the CVT, the control appraisal refers to how participants perceive they are in control of their learning. Moreover, these items are not clearly separated from other constructs such as self-efficacy. A similar point has to be raised regarding the items used to measure value. Although they fit with the definition of “value” in both theories, they may also measure related concepts such as test anxiety (“the risk of failure scares me”) or grade goals (“it is important for me to get a good grade on the exams”). A third limitation of our results is that they are purely correlational; all data were collected at the same time and we cannot provide any conclusion regarding causality. Finally, data were collected during the first COVID-19 lockdown, which is a very particular context—especially during the first lockdown of 2020. The risk of dropping out of school during the pandemic may be higher, particularly because of online courses [41,42]. Indeed, students often reported that they found it very difficult to stay focused, and thus may have felt more bored, during online teaching. Future studies should test this hypothesis. However, while dropout levels may be higher, we hypothesize that the mechanisms at work may be the same. Thus, our results suggest that by influencing the control that students have over their studies, it is possible to reduce dropouts, which in the current context is a major issue for training programs.

Note that we asked participants to respond about their perception of their academic training in general. It is possible that perceptions of boredom in the curriculum vary between learning situation and courses. It is also possible that boredom has a different relationship with the risk of dropping out depending on different courses studied. The aim of this study was to have a global view of the phenomenon but future research could take into account potential differences in perceptions between learning situations. Furthermore, future work should consider both proximal and distal antecedents of boredom. Notably, they could adopt another line of analysis by integrating participants’ tendency towards depression or other psychiatric symptoms such as Attention Deficit Hyperactivity Disorder (ADHD). As these may be related to boredom, they could provide interesting insights in distal antecedents of boredom. Finally, future studies should collect longitudinal data in order to provide causal inference regarding the impact of control and value on boredom as well as on the intention to persist. 

## 5. Conclusions

To conclude, our results highlight the importance of students’ perception of control and value on the emergence of boredom, which in turn is related to their persistence in their studies. In the specific context of teacher education, this emphasizes how crucial it is to understand and to help pre-service teachers cope with boredom in their studies. Our results notably suggest that students need to feel that they have control over their studies, and they further need to value them, so that the potentially detrimental impact of boredom on the intention to persist may be contained.

## Figures and Tables

**Table 1:** Descriptive statistics and variable correlations.

	M.	S.D.	1.	2.	3.	4.
1. Control	4.98	1.22	1	0.03	−0.26 ***	0.40 ***
2. Value	5.77	1.07		1	−0.10.	0.05
3. Boredom	4.08	1.25			1	−0.27 ***
4. Intention to persist	5.93	1.10				1

Note. M = mean; S.D. = standard deviation. Scales range from 1 to 7, *** *p* < 0.001.

**Table 2:** Factors loading.

	Unstandardized Estimate	Standardized Estimate	S.E.	z	*p*-Value	95% CI
Control						
C1	1.00	0.82	0.04	21.96	*p* < 0.001	[0.74; 0.89]
C2	0.77	0.63	0.05	13.42	*p* < 0.001	[0.54; 0.73]
C3	1.00	0.83	0.04	22.23	*p* < 0.001	[0.75; 0.91]
Value						
V1	1.00	0.76	0.05	16.38	*p* < 0.001	[0.67; 0.85]
V2	0.84	0.79	0.04	21.22	*p* < 0.001	[0.71; 0.86]
V3	0.78	0.60	0.06	10.27	*p* < 0.001	[0.48; 0.72]
V4	0.35	0.40	0.07	5.67	*p* < 0.001	[0.25; 0.53]
Intention to persist						
P1	1.00	0.83	0.03	28.66	*p* < 0.001	[0.77; 0.89]
P2	1.04	0.72	0.05	15.60	*p* < 0.001	[0.63; 0.81]
P3	0.83	0.46	0.05	90.08	*p* < 0.001	[0.36; 0.56]
P4	1.00	0.76	0.04	17.76	*p* < 0.001	[0.68; 0.84]
P5	0.77	0.69	0.05	13.08	*p* < 0.001	[0.59; 0.79]
P6	0.76	0.58	0.06	9.18	*p* < 0.001	[0.45; 0.70]
P7	0.64	0.70	0.06	11.43	*p* < 0.001	[0.58; 0.82]
P8	0.78	0.57	0.06	10.27	*p* < 0.001	[0.46; 0.69]
Boredom						
B1	1.00	0.74	0.07	10.51	*p* < 0.001	[0.60; 0.87]
B2	0.73	0.47	0.07	7.16	*p* < 0.001	[0.34; 0.60]
B3	0.73	0.55	0.07	8.03	*p* < 0.001	[0.42; 0.69]

**Table 3:** Regression coefficients between factors.

	Unstandardized Estimate	Standardized Estimate	*S.E.*	z	*p*-Value	95% CI
Control ↔ Value	0.02	0.03	0.08	0.40	0.69	[−0.12; 0.18]
Control ↔ Intention to persist	0.39	0.38	0.07	6.03	*p* < 0.001	[0.26; 0.51]
Value ↔ Intention to persist	0.21	0.08	0.06	1.21	0.22	[−0.05; 0.20]
Control ↔ boredom	−0.34	−0.33	0.08	−4.32	*p* < 0.001	[−0.48; −0.18]
Value ↔ boredom	−0.37	−0.13	0.08	−1.70	0.09	[−0.29; 0.02]
Boredom ↔ Intention to persist	−0.18	−0.18	0.07	−2.76	0.01	[−0.31; −0.05]

**Table 4:** Indirect and direct effects.

	Unstandardized Estimate	Standardized Estimate	*S.E.*	z	*p*-Value	95% CI
Control → Boredom → Intention to persist	0.06	0.06	0.03	2.21	0.03	[0.01; 0.11]
Value → Boredom → Intention to persist	0.07	0.02	0.01	1.61	0.11	[−0.01; 0.05]
Total effect	0.72	0.54	0.08	6.83	*p* < 0.001	[0.39; 0.70]

## Data Availability

The data presented in this study are available on request from the corresponding author.
